# Temperature-Triggered Switchable Helix-Helix Inversion of Poly(phenylacetylene) Bearing l-Valine Ethyl Ester Pendants and Its Chiral Recognition Ability

**DOI:** 10.3390/molecules21111583

**Published:** 2016-11-21

**Authors:** Yanli Zhou, Chunhong Zhang, Yuan Qiu, Lijia Liu, Taotao Yang, Hongxing Dong, Toshifumi Satoh, Yoshio Okamoto

**Affiliations:** 1 Polymer Materials Research Center, Key Laboratory of Superlight Materials and Surface Technology, Ministry of Education, College of Materials Science and Chemical Engineering, Harbin Engineering University, Harbin 150001, China; zhouyanli0808022@163.com (Y.Z.); wukong.s@163.com (Y.Q.); az106321@126.com (T.Y.); dhongxing@hrbeu.edu.cn (H.D.); okamoto@apchem.nagoya-u.ac.jp (Y.O.); 2 Faculty of Engineering, Hokkaido University, Sapporo 060-8628, Japan; satoh@eng.hokudai.ac.jp; 3 Graduate School of Engineering, Nagoya University, Furo-cho, Chikusa-ku, Nagoya 464-8601, Japan

**Keywords:** helical poly(phenylacetylene), temperature-triggered, helix-helix inversion, chiral stationary phases, *cis*-to-*trans* isomerization

## Abstract

A phenylacetylene containing the l-valine ethyl ester pendant (PAA-Val) was synthesized and polymerized by an organorhodium catalyst (Rh(nbd)BPh_4_) to produce the corresponding one-handed helical *cis*-poly(phenylacetylene) (PPAA-Val). PPAA-Val showed a unique temperature-triggered switchable helix-sense in chloroform, while it was not observed in highly polar solvents, such as *N*,*N*′-dimethylformamide (DMF). By heating the solution of PPAA-Val in chloroform, the sign of the CD absorption became reversed, but recovered after cooling the solution to room temperature. Even after six cycles of the heating-cooling treatment, the helix sense of the PPAA-Val’s backbone was still switchable without loss of the CD intensity. The PPAA-Val was then coated on silica gel particles to produce novel chiral stationary phases (CSPs) for high-performance liquid chromatography (HPLC). These novel PPAA-Val based CSPs showed a high chiral recognition ability for racemic mandelonitrile (*α* = 2.18) and racemic *trans*-*N*,*N*′-diphenylcyclohexane-1,2-dicarboxamide (*α* = 2.60). Additionally, the one-handed helical *cis*-polyene backbone of PPAA-Val was irreversibly destroyed to afford PPAA-Val-H by heating in dimethyl sulfoxide (DMSO) accompanied by the complete disappearance of the Cotton effect. Although PPAA-Val-H had the same l-valine ethyl ester pendants as its *cis*-isomer PPAA-Val, it showed no chiral recognition. It was concluded that the one-handed helical *cis*-polyene backbone of PPAA-Val plays an important role in the chiral recognition ability.

## 1. Introduction

Biomacromelocules, such as DNA and proteins, possess helical structures, which are known to play key roles in nature and closely linked with specific activities in living systems [1,2]. Inspired by nature, a number of helical polymers, including polyisocyanates [3,4], polyisocyanides [5,6,7], polytriarylmethyl methacrylates [8,9], etc. [10,11,12], have been synthesized and their properties widely studied. One of the unique features of helical polymers is the helix-helix transition from the one helical conformation to another which is induced by external stimuli, including solvent, salt concentration, pH, temperature, or light irradiation. These switchable helical polymers have broad applications in liquid crystals, data storage, and switching devices [10,13]. *cis*-Poly(phenylacetylene) prepared by an organorhodium catalyst is a typical dynamic helical polymer [14]. Their helix-helix inversions based on the dynamic *cis*-backbone were studied by Yashima, Freire, and other groups [15]. Generally, to realize this helix-helix inversion, an extraneous small molecule was necessary. However, only a few studies have reported results regarding the switchable endogenous helix-helix inversion of polyacetylene without the assistance of extraneous molecules.

Another attractive feature of optically-active poly(phenylacetylene)s is their chiral recognition ability as chiral stationary phases (CSPs) for high-performance liquid chromatography (HPLC). Some high stereoregular helical poly(phenylacetylene)s bearing chiral pendants, synthesized utilizing an organorhodium catalyst, have proved to be prospective CSPs for the HPLC separation of enantiomers [16,17,18,19,20]. Amino acids are naturally-occurring building block and have been widely applied in polymer science. Kakuchi reported an anion sensor based on poly(phenylacetylene)s bearing the l-leucine methyl ester [21]. Tang investigated the hierarchical structures of amphiphilic poly(phenylacetylene)s bearing l-valine pendants [22]. In our previous studies, we reported that the CSP based on poly(phenylacetylene)s bearing amino acid pendants, such as l-phenylalanine, l-phenylglycine and l-leucine pendants, that showed good chiral recognition abilities [19,20,23]. It was determined that as the linkage group between the polyene main chain and the chiral pendant amide is effective for improving the chiral recognition of the poly(phenylacetylene)s; the polymers having an amide linkage group showed a higher chiral recognition than the ones having urea or sulfonamide linkage groups [19,23]. However, there exist no reports regarding the chiral recognition ability of poly(phenylacetylene)s bearing l-valine pendants as CSPs, and the chiral recognition mechanism of these helical poly(phenylacetylene)s bearing amino acid pendants as CSPs, especially the contributions of their one-handed helical *cis*-polyene backbone to the chiral recognition ability, is still not very clear.

In this study, a novel one-handed helical *cis*-poly(phenylacetylene), in which the l-valine ethyl ester pendants are linked with the polyene main chain by an amide group (PPAA-Val), was designed and synthesized, and the effects of solvent and temperature on their helical structure and CD signals are discussed. To evaluate the chiral recognition ability of PPAA-Val and its potential for chiral separation, PPAA-Val was coated on silica gel particles to produce a novel type of CSP for HPLC and a batch of chiral separations occurred on these CSPs. In addition, PPAA-Val was transformed into PPAA-Val-H by heat treatment [24,25,26,27,28,29,30], and the contribution of the one-handed helical *cis*-structure of PPAA-Val for its chiral recognition was discussed.

## 2. Results and Discussion

### 2.1. Synthesis and Polymerization of PAA-Val

**Scheme 1:**
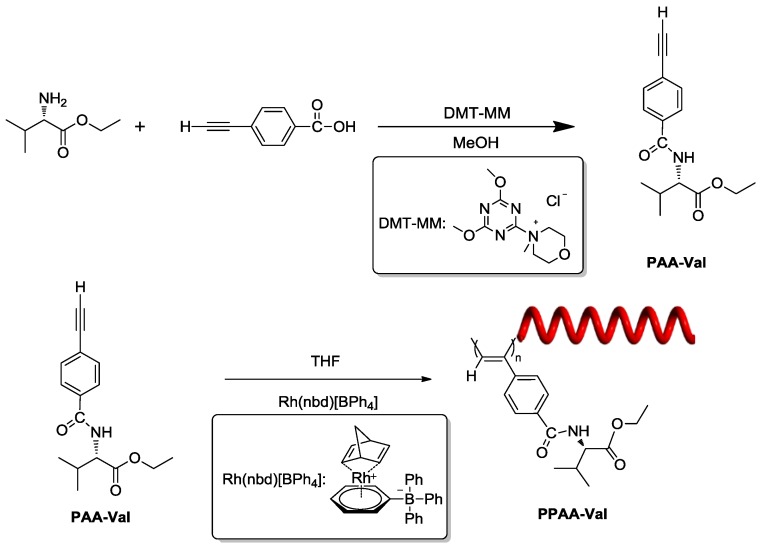
Synthesis of poly(phenylacetylene) derivative PPAA-Val.

**Figure 1:**
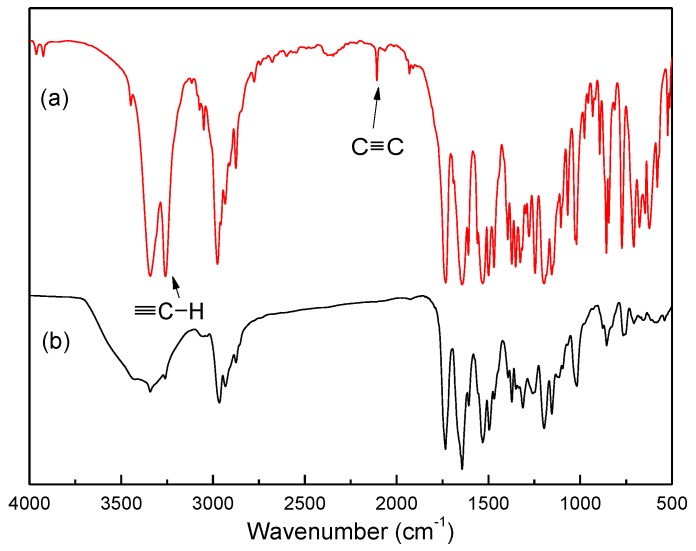
IR spectra of (**a**) monomer PAA-Val and (**b**) its polymer PPAA-Val.

**Figure 2:**
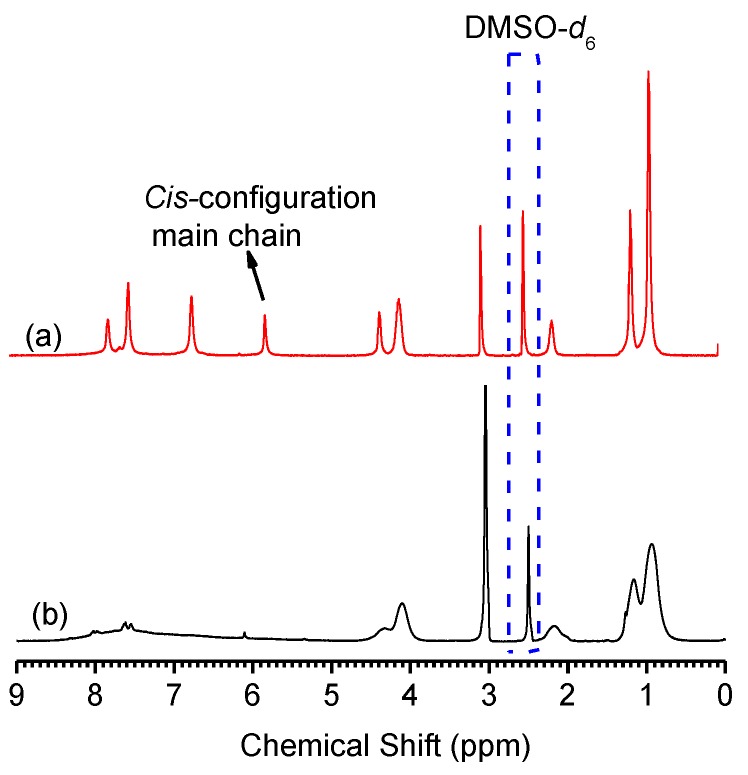
^1^H-NMR spectra of (**a**) PPAA-Val and (**b**) PPAA-Val-H in DMSO-*d*_6_ at 80 °C (the polymers are soluble in DMSO-*d*_6_ at 80 °C).

Scheme 1 illustrates the synthetic and polymerization routes for PAA-Val. The amidation reaction of 4-ethynylbenzoic acid with the l-valine ethyl ester proceeded by using 4-(4,6-dimethoxy-1,3,5-triazin-2-yl)-4-methylmorpholinium chloride (DMT-MM) as a condensation agent in a good yield to produce the novel phenylacetylene, PAA-Val. The polymerization of PAA-Val was carried out in tetrahydrofuran (THF) at room temperature for 24 h using Rh(nbd)BPh_4_ as the catalyst to produce PPAA-Val as a yellow solid in 76.8% yield. The *M*_n_ and *M*_w_/*M*_n_ of PPAA-Val were 1.1 × 10^5^ and 2.23, respectively. PPAA-Val was soluble in CHCl_3_, MeOH, acetone, THF, *N*,*N*′-dimethylformamide (DMF), *N*,*N’*-dimethylacetamide (DMAc), and partially soluble in dimethyl sulfoxide (DMSO) at room temperature. As shown in Figure 1, the absorption bands at 3259 and 2108 cm^−1^ associated with the ≡C-H and C≡C stretching vibrations of PAA-Val were not observed in the spectra of its polymer (PPAA-Val) (Figure 1b), suggesting that the acetylene triple bond of PAA-Val was consumed by the polymerization. The ^1^H-NMR spectrum of PPAA-Val showed the characteristic signal of the *cis*-proton at 5.75 ppm, indicative of the propensity of the *cis*-configuration in the poly(phenylacetylene) main chain (Figure 2a) [21].

### 2.2. Secondary Structure of PPAA-Val

The specific rotations ([$\alpha_{D}^{20}$]) of PAA-Val and its polymer PPAA-Val in various solvents at room temperature are summarized in Table 1. The [$\alpha_{D}^{20}$] values of PPAA-Val were much higher than those of its corresponding monomer in all the solvents; e.g., the [$\alpha_{D}^{20}$] of PPAA-Val in MeOH (+334°) was thirty-fold greater than that of PAA-Val (+10.3°). This means that the high optical activity of PPAA-Val is not only from its chiral pendants. Since a one-handed helical polymer chain can exhibit a very high optical activity [31,32], the one-handed helicity in the polymer is suspected to be the reason for the higher optical activity of the polymer.

**Figure 3:**
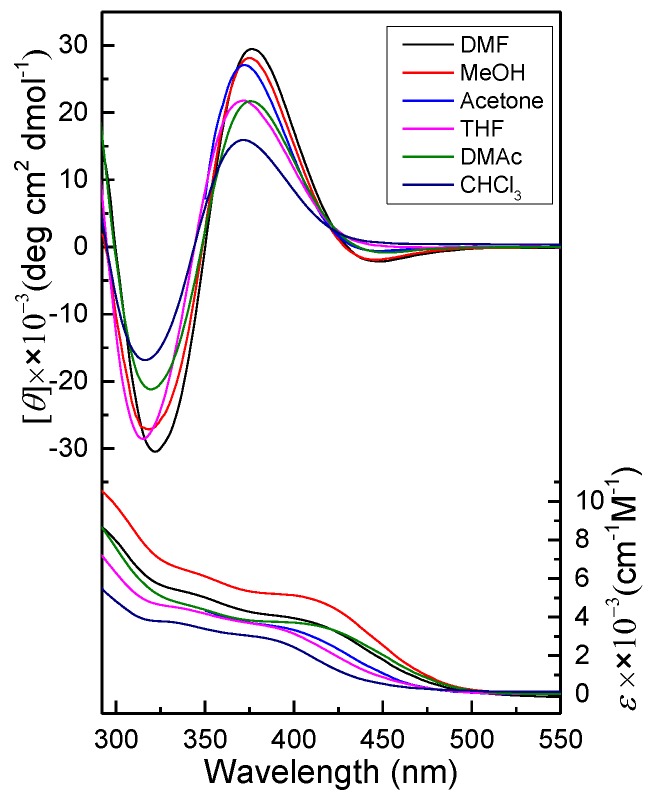
CD (upper) and UV-Vis (lower) spectra of PPAA-Val in variable solvents at 25 °C (*c* = 1 mg/mL).

To confirm the helical structure of the PPAA-Val, its chiroptical properties were investigated in different solvents at 25 °C by CD spectroscopy (Figure 3). The polymer showed similar split Cotton effects in MeOH, acetone, THF, DMF, DMAc, and CHCl_3_ in the range from 300–500 nm, i.e., the first positive Cotton effect at 375 nm and the second negative Cotton effect at 320 nm, indicating the one-handed helical main chain of PPAA-Val [33]; in other words, the one-handed helical PPAA-Val bearing l-valine ethyl ester pendants was successfully synthesized.

The induced helical sense in the backbone of PPAA-Val by the chiral pendants was thought to be maintained by the intramolecular hydrogen bonding and steric repulsion between the chiral side chains [34]. Generally, a highly polar solvent can weaken the intra- and/or inter- molecular hydrogen bonds, and decrease the stability of the induced helicity in the polymer [35,36]. However, PPAA-Val showed stronger CD absorptions in the highly polar solvents, such as DMF and methanol, than those in chloroform and THF at 25 °C. It is possible that the interior stress in the polymer may relax in the highly polar solvents which can weaken the intramolecular hydrogen bonding between the adjacent side chains.

These speculations have been supported by the FT-IR and UV analyses. The intramolecular hydrogen bonding in PPAA-Val was investigated based on the IR spectra of PAA-Val and PPAA-Val in the solid- and liquid-states (Table 2). In the solid state, both PAA-Val and PPAA-Val exhibited the amide ν_C=O_ at 1644 cm^−1^, indicating that the hydrogen-bonded amide groups were formed. In CHCl_3_, PPAA-Val exhibited the ν_C=O_ of the amide group at 1648 cm^−1^, which was 14 cm^−1^ lower than that of PAA-Val (1662 cm^−1^). On the other hand, PAA-Val and PPAA-Val exhibited the ν_C=O_ of the ester group at a similar wavenumber in both the solid state and their solution (Table 2). These results suggested that, in CHCl_3_, the intramolecular hydrogen bonding was formed between the amide groups in the valine ethyl ester side chains, while the ester group had no contribution to the hydrogen bonding. The UV-VIS absorptions of PPAA-Val in different solvents are shown in Figure 3. In DMF and MeOH, the absorption of the polyene backbone of PPAA-Val appeared at a higher wavelength (around 420 nm) than those in CHCl_3_ (around 390 nm). This may be associated with the fact that the longer conjugated length in the polyene backbone in the more polar solvent is shortened by the intramolecular hydrogen bonding in the polymer than in a nonpolar solvent [37].

Hence, in chloroform, the intramolecular hydrogen bonding in the polymer may disturb the chiral induction to the main chain by the chiral valine ethyl ester pendants and may cause the lower regularity of the helical structure of PPAA-Val. In the more polar solvents, the interior stress in the polymer is relaxed due to the breaking of the intramolecular hydrogen bonding; therefore, the chirality in the polyene backbone can be efficiently induced to afford the stronger CD absorptions of PPAA-Val.

### 2.3. Temperature-Triggered Helix-Helix Inversion of PPAA-Val

**Figure 4:**
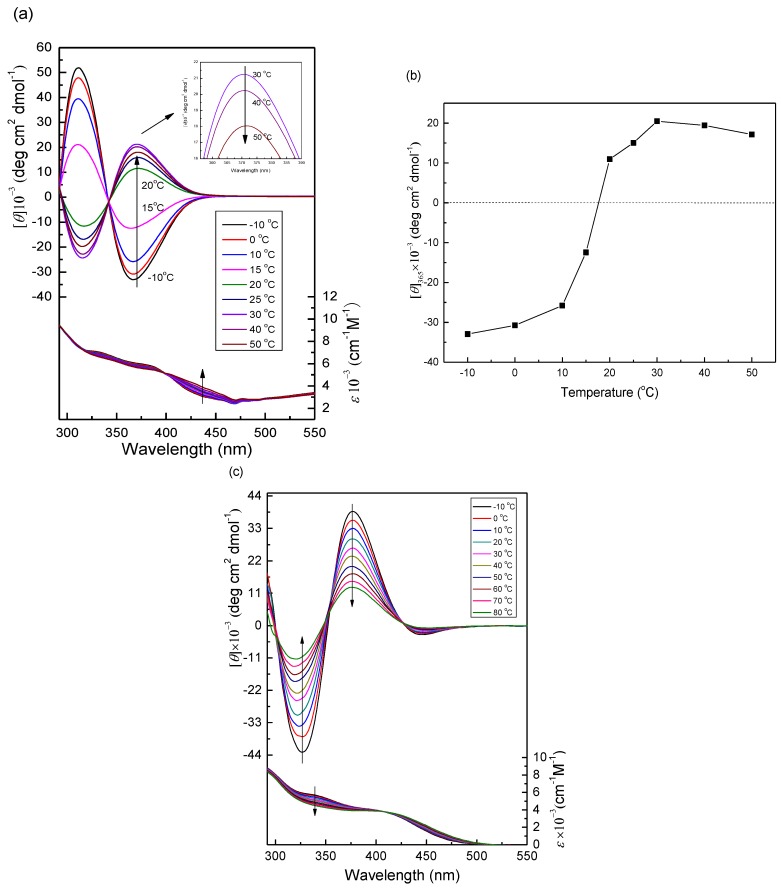
Temperature dependence of CD and UV-VIS spectra of PPAA-Val in CHCl_3_ (**a**); plots of [*θ*]_365_ of PPAA-Val in CHCl_3_ versus temperature (**b**); and CD and UV-VIS spectra of PPAA-Val in DMF at different temperature (**c**) (*c* = 1 mg/mL).

**Figure 5:**
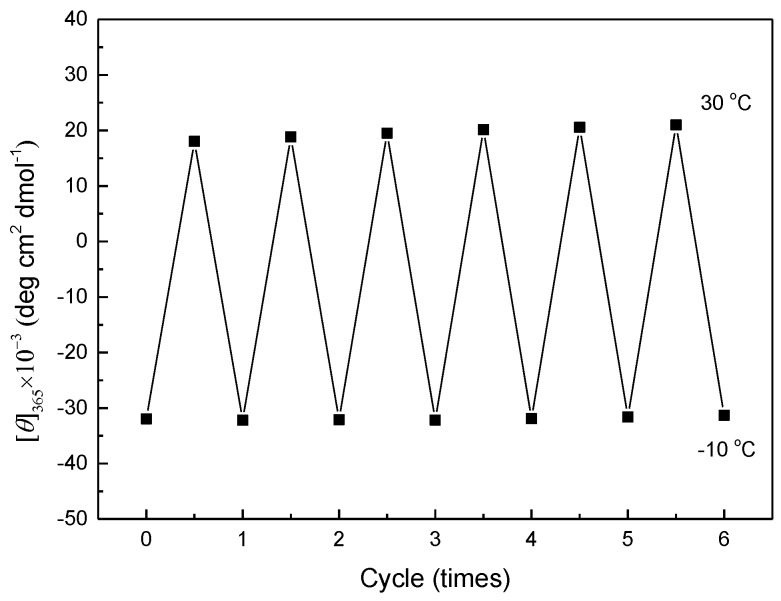
Plots of the [*θ*]_365_ values of PPAA-Val in chloroform upon continuous thermocycling (−10–30 °C) in CHCl_3_ (*c* = 1 mg/mL).

To investigate the thermostability of the helix sense of PPAA-Val in different solvents, the CDs of PPAA-Val were separately measured in CHCl_3_ and DMF at different temperatures (Figure 4). Interestingly, in chloroform, PPAA-Val showed CD signals with opposite signs at the low and high temperatures. At −10 °C, PPAA-Val showed a negative first Cotton effect at around 365 nm and a positive second Cotton effect at a wavelength around 310 nm, and the signs of these CD signals were opposite to the CD signals measured in DMF (Figure 3 and Figure 4). With the increasing temperature, the CD intensities initially decreased, then the CD signals became inverted to the ones with the opposite signs, and the CD signals’ pattern at high temperature became the same as the CD in DMF (Figure 5a,b). Figure 4b shows the temperature dependence of the first Cotton effect at 365 nm in chloroform. From −10–10 °C, the intensity of the negative [*θ*]_365_ value gradually decreased, then sharply inverted to be positive from 10–30 °C. The positive [*θ*]_365_ value then slightly decreased by further heating the solution to 50 °C. Since the Cotton effect around 365 nm is due to the one-handed helical main chain conformation of PPAA-Val, the inversion of the CD from −10–30 °C in chloroform indicates the inversion of the helix-sense of the main chain. In addition, this temperature-triggered helix-helix inversion in the polyene main chain of PPAA-Val was reversible and switchable, even after six cycles of thermocycling (−10–30 °C) (Figure 5). In summary, a temperature-triggered switchable helix-helix inversion of PPAA-Val was realized in chloroform without the assistance of any other extraneous compounds. However, this interesting temperature-triggered inversion of the CD signals of PPAA-Val was not observed in DMF, and the CD intensity only showed a one-way decrease in DMF with the increasing temperature (Figure 4c).

Generally, hydrogen bonding is strengthened at low temperature and weakened at high temperature [35,38,39]. Compared to the relaxed structure of PPAA-Val in DMF due to the weakened intramolecular hydrogen bonding, in chloroform, the low temperature strengthened the intramolecular hydrogen bonding and caused the tight hydrogen-bonding strands of the side chains in the polymer. These might afford the different spatial positions of chiral pendants and the opposite helix sense compared to those in DMF. With the increasing temperature, the intramolecular hydrogen bonding was weakened and the inner stress in the polymer chain was relaxed, so that the spatial positions of the side chains and the main chain helix recovered to be the same as the relaxed state in DMF. When the solution was cooled again, the intramolecular hydrogen bonds recovered and the helix inverted to be the same as the original direction. This is a unique temperature-triggered helix-helix inversion based on the dynamic polyene backbone of PPAA-Val without the co-existence of any other extraneous molecules. The one-way decrease of the CD in DMF with the increasing temperature should be caused by the violent thermo motion of the molecules at high temperature, which caused the irregularity of the polymer’s helical structure.

### 2.4. Chiral Recognition Ability of PPAA-Val

**Figure 6:**
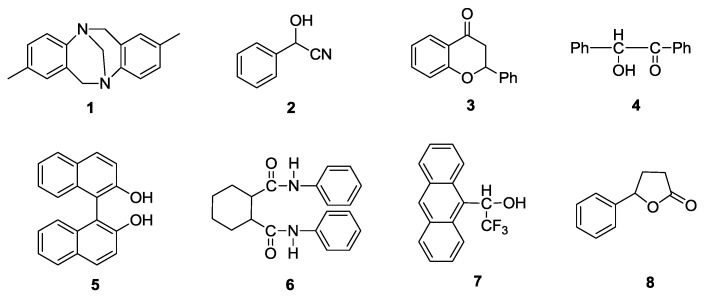
Structures of racemates.

**Figure 7:**
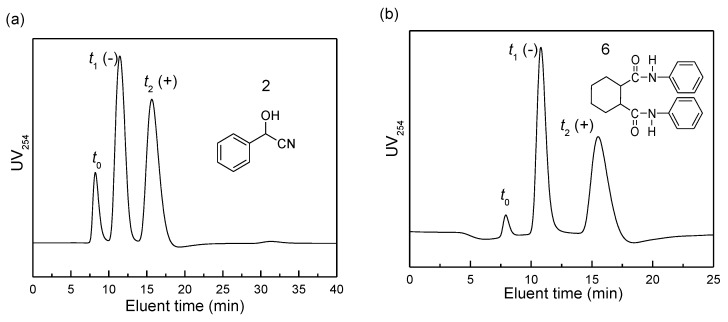
Chromatograms for the resolution of (**a**) **2** and (**b**) **6** on PPAA-Val coated with DMF with hexane/2-propanol (90/10) as eluent.

Since PPAA-Val not only has chiral pendants, but also a one-handed helical polyene backbone, the polymer was expected to be effective for chiral recognition. Its chiral recognition ability was evaluated as CSPs by HPLC using the eight tested racemates (Figure 6). Since PPAA-Val showed different chiroptical properties in different solvents, DMF, THF, and CHCl_3_ were separately selected as the coating solvent to coat the PPAA-Val on silica gel. The influences of the coating solvents on the resolutions of the racemates on PPAA-Val were evaluated by HPLC, and the coating solvents clearly influenced the chiral recognition abilities of the PPAA-Val-based CSPs (Table 3). Figure 7 shows the chromatograms for racemates **2** and **6** by the CSP coated with DMF, and their enantiomers are completely separated with the high separation factors of 2.18 for **2** and 2.60 for **6**. Therefore, a novel CSP based on the one-handed helical poly(phenylacetylene) having l-valine ethyl ester pendants has been successfully prepared and showed a good chiral recognition ability in HPLC. However, our previous CSPs based on the helical poly(phenylacetylene)s bearing l-phenylalanine, l-phenylglycine or l-leucine pendants did not show any chiral recognition for racemates **2** and **6**, although they showed chiral recognitions for the other racemates [19,20,23]. The novel PPAA-Val-based CSPs can be complementary with the our previous poly(phenylacetylene)s-based CSPs.

In addition, the chiral recognition abilities of the PPAA-Val-based CSPs coated with THF and CHCl_3_ were lower than the CSPs coated with DMF (Table 3). The higher chiral recognition ability of the CSP coated with DMF should be correlated to the stronger Cotton effects, i.e., the higher one-handedness of its *cis*-backbone of the polymer in DMF at the coating temperature (25 °C), and this more one-handed helical structure may be retained in the CSP which may result in the higher chiral recognition ability.

### 2.5. Effects of One-Handed Helical cis-Polyene Main Chain of PPAA-Val on its Chiral Recognition Ability

To investigate the influence of the regularity and configuration of the helical polyene backbone in poly(phenylacetylene) on their chiroptical properties and chiral recognition ability, a DMSO solution of PPAA-Val was heated to 150 °C for 10 min under nitrogen to destroy its one-handed helical *cis*-polyene backbone to afford PPAA-Val-H. After the heat treatment, the color of the polymer solution changed from orange to red brown. In the ^1^H-NMR of the PPAA-Val, the signal of the *cis*-proton in PPAA-Val at 5.75 ppm disappeared after the heat treatment, and the sharp ^1^H-NMR signals of the main chain became broader along with a decreasing peak intensity (Figure 2) [40,41]. These results suggest that during heat treatment, the one-handed helical *cis*-polyene backbone of PPAA-Val collapsed. The *M*_n_ and *M*_w_/*M*_n_ of PPAA-Val-H were 3.2 × 10^4^ and 1.98, respectively. The decreased *M*_n_ (from 1.1 × 10^5^ to 3.2 × 10^4^) indicates that degradation of the polymer occurred by the heat treatment [28].

**Figure 8:**
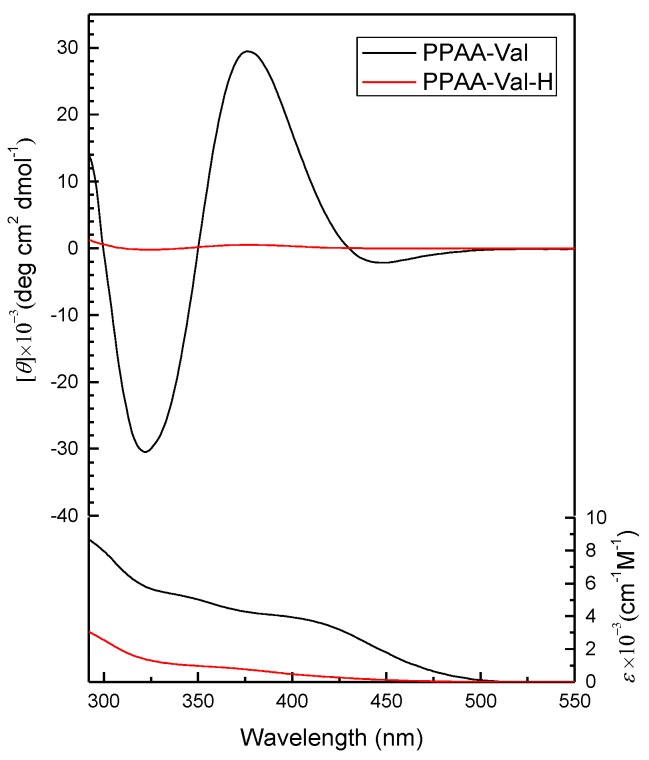
CD and UV-VIS spectra of PPAA-Val and PPAA-Val-H measured in DMF at 25 °C (*c* = 1 mg/mL).

After the heat treatment of PPAA-Val with the *cis*-polyene backbone, the CD absorptions completely disappeared, although the polymers had the same chiral pendants. This indicated the preferred one-handed helical structure of PPAA-Val irreversibly collapsed after the heat treatment. As shown in the lower part of Figure 8, the broad band up to 500 nm due to the π-π* transition of the main chain of PPAA-Val weakened after the heat treatment. Therefore, it is concluded that the weakened absorption should be caused by the cleavage or deflection of the main chain and PPAA-Val-H lost its coplanar property of the conjugated double bonds [28,42].

The chiral recognition ability of the CSP coated by PPAA-Val-H with DMF was also evaluated using the tested racemates by HPLC, however, the PPAA-Val-H based CSP had no recognition ability for all the tested racemates although it took the same chiral pendants with the *cis*-PPAA-Val (Table 3). These results indicated that as the CSP, the regular one-handed helical *cis*-polyene backbone of PPAA-Val played key roles in the chiral recognition by HPLC. In summary, a regular helical *cis*-backbone is essential for the high chiral recognition ability of the poly(phenylacetylene) derivatives.

## 3. Materials and Methods

### 3.1. Materials

l-Valine (purity 99%) and 4-(4,6-dimethoxy-1,3,5-triazin-2-yl)-4-methylmorpholinium chloride (DMT-MM) (purity 98%) were purchased from Sahn Chemical Technology Co., Ltd. (Shanghai, China). Hydrochloride in ethanol (30%–40%) was purchased from Chengdu Xiya Chemistry Technology Co., Ltd. (Chengdu, China). Triphenylphosphine (purity 99%) was purchased from J and K Chemical Co., Ltd. (Beijing, China). 4-Ethynylbenzoic acid was synthesized according to a previously reported method [43]. Rh^+^(2,5-norbornadiene)((*η*^6^-C_6_H_5_)B(C_6_H_5_)_3_) [Rh(nbd)BPh_4_] was prepared based on a previous report [44]. All of the solvents used in the reactions were of analytical grade, carefully dried, and distilled before use. Silica gel with a mean particle size of 37–56 μm for column chromatography was purchased from Qingdao Haiyang Chemical Co., Ltd. (Qingdao, China). The porous spherical silica gel with a mean particle size of 7 μm and a mean pore diameter of 100 nm (Daiso gel SP-1000-7) for HPLC was kindly supplied by Daiso Chemicals (Osaka, Japan), then silanized with (3-aminopropyl)triethoxysilane in toluene at 80 °C before use. All of the solvents used in the preparation of the chiral stationary phases were of analytical grade. Hexane and 2-propanol used in chromatographic experiments were of HPLC grade. The racemates were commercially available or were prepared by the usual methods.

### 3.2. Instruments

The ^1^H- and ^13^C-NMR spectra (500 MHz) were recorded using a Bruker AVANCE III-500 (Fällanden, Switzerland) instrument at room temperature. The IR spectra were obtained using a Perkin-Elmer FTIR-100 spectrophotometer (Fremont, CA, USA). The number-average molecular weight (*M*_n_), the weight-average molecular weight (*M*_w_), and the polydispersity (*M*_w_/*M*_n_) of the polymers were determined by size exclusion chromatography (SEC) calibrated with standard polystyrenes at 40 °C using a JASCO SEC system (Tokyo, Japan) (PU-980 Intelligent pump, CO-965 column oven, RI-930 Intelligent RI detector, and Shodex (Tokyo, Japan) DEGAS KT-16) equipped with a Shodex Asahipak GF-310 HQ column (linear, 7.6 mm × 300 mm; pore size, 20 nm; bead size, 5 μm; exclusion limit, 4 × 10^4^) and a Shodex Asahipak GF-7 M HQ column (linear, 7.6 mm × 300 mm; pore size, 20 nm; bead size, 9 μm; exclusion limit, 4 × 10^7^) in DMF containing lithium chloride (0.01 M) at the flow rate of 0.4 mL/min. The optical rotation was measured at room temperature using a Perkin-Elmer Model 341 polarimeter (Fremont, CA, USA). The circular dichroism (CD) and ultraviolet visible (UV-VIS) spectra were measured in a 1-mm path length cell using a JASCO J-815 spectropolarimeter (Tokyo, Japan). All the enantioseparation experiments were performed using a JASCO PU-2089 high performance liquid chromatograph (HPLC) system (Tokyo, Japan) equipped with UV-VIS (JASCO-UV-2070) and circular dichroism (JASCO-CD-2095) detectors. A solution of a racemate (3 mg/mL) was injected into the chromatographic system through an intelligent sampler (JASCO AS-2055).

### 3.3. Synthesis of l-Valine Ethyl Ester

The l-valine ethyl ester was synthesized via the esterification reaction of the l-valine in a hydrochloride ethanol solution. A typical procedure is described as follows: l-Valine (8.00 g, 68.0 mmol) and hydrochloride in ethanol (1.50 mol/L, 160 mL) were added to a 500 mL round-bottomed flask. The mixture was refluxed with stirring for 12 h and turned yellow. It was then cooled to room temperature, and extracted with a saturated NaHCO_3_ aqueous solution and DCM. The organic layer was dried using MgSO_4_ and filtered. The filtrate was evaporated to remove the DCM to give the l-valine ethyl ester as a brown-yellow liquid. Yield: 7.63g (81.5%). ^1^H-NMR (500 MHz, CDCl_3_, TMS, ppm): δ = 4.20-4.00 (q, 2H, -C***H***_2_-O-), 3.17–3.10 (d, 1H, -C***H***-NH_2_), 2.00–1.85 (m, 1H, -C***H***-(CH_3_)_2_), 1.35 (s, 2H, -N***H***_2_), 1.20–1.10 (t, 3H, -O-CH_2_-C***H***_3_), 0.88–0.79 (dd, 6H, -CH-(C***H***_3_)_2_).

### 3.4. Synthesis of N-(4-Ethynylbenzoyl)-l-Valine Ethyl Ester (PAA-Val)

The *N*-(4-ethynylbenzoyl)-l-valine ethyl ester (PAA-Val) was synthesized via the amidation reaction between 4-ethynylbenzoic acid and l-valine ethyl ester. A typical procedure is described as follows: To a solution of the 4-ethynylbenzoic acid (3.85 g, 26.3 mmol) and l-valine ethyl ester (3.82 g, 26.3 mmol) in MeOH (132 mL) was added the DMT-MM (7.99 g, 28.9 mmol). After stirring at room temperature for 18 h, the reaction mixture was purified by column chromatography on silica gel with hexane/ethyl acetate (3/1, *v*/*v*) to give PAA-Val as a white solid. Yield: 5.36 g (74.5%). ^1^H-NMR (500 MHz, CDCl_3_, TMS, ppm): δ = 7.80–7.76 (d, 2H, Ar-***H***), 7.54–7.52 (d, 2H, Ar-***H***), 6.80–6.79 (d, 1H, -N***H***-), 4.78–4.70 (q, 1H, NH-C***H***-), 4.35–4.15 (q, 2H, -O-C***H***_2_-), 3.24 (s, 1H, ≡C***H***), 2.29–2.20 (m, 1H, -C***H***-(CH_3_)_2_), 1.40–1.20 (t, 3H, -O-CH_2_-C***H***_3_), 1.02–0.85 (dd, 6H, -CH-(C***H***_3_)_2_). ^13^C-NMR (125 MHz, CDCl_3_, TMS, ppm): δ = 174.5 (-CO- (amido)), 155.0 (-CO-(ester)), 138.7 (aromatic), 132.5 (aromatic), 116.9 (aromatic), 113.0 (aromatic), 82.6 (-C≡CH), 78.2 (-C≡CH), 61.4(-CH_2_-CH_3_), 52.6 (-CH-NH), 24.5 (-CH), 22.3 (-CH_3_), 21.7 (-CH_3_), 14.2 (-CH2-CH_3_). IR (cm^−1^, KBr): 3342 (N-H), 3259 (≡C-H), 2108 (C≡C), 1733 (C=O), 1644 (C=O).

### 3.5. Polymerization

The polymerization of PAA-Val was carried out in a dry Schlenk flask under a dry nitrogen atmosphere using Rh(nbd)BPh_4_ as the catalyst in similar way as previously reported [19,21,45]. A typical procedure is described as follows: PAA-Val (2.00 g, 7.32 mmol) was weighed into a Schlenk flask and dissolved in dry THF (220 mL) before a solution of Rh(nbd)BPh_4_ (75.3 mg, 146 μmol) in dry tetrahydrofuran (THF, 24.0 mL) was added. After stirring at room temperature for 24 h, triphenylphosphine (75.3 mg, 0.292 mmol) was added to the reaction mixture. The solution was concentrated, then poured into a large amount of hexane (2000 mL). The precipitates were purified by reprecipitation using hexane, then dried under reduced pressure to give PPAA-Val as a yellow solid (1.54 g, 76.8%). *M*_n_ = 1.10 × 10^5^; *M*_w_/*M*_n_ = 2.23. ^1^H-NMR (500 MHz, DMSO-*d*_6_, TMS, ppm): δ = 7.74 (d, 1H, -N***H***-), 7.48 (d, 2H, Ar-***H***), 6.68 (d, 2H, Ar-***H***), 5.75 (s, 1H , main chain), 4.30 (q, 1H, NH-C***H***-), 4.05 (q, 2H, -O-C***H***_2_-), 2.11 (m, 1H, -C***H***-(CH_3_)_2_), 1.11 (t, 3H, -CH_2_-C***H***_3_), 0.88 (dd, 6H, -CH-(C***H***_3_)_2_). IR (cm^−1^, KBr): 3341 (N-H), 1734 (C=O), 1644 (C=O).

### 3.6. Heat Treatment of PPAA-Val

A typical procedure is described as follows: In a nitrogen atmosphere, PPAA-Val (0.3 g) was weighed into a flask and partially dissolved in dry dimethyl sulfoxide (DMSO, 30 mL). After stirring at 150 °C for 10 min, the mixture was cooled to room temperature, then the solution was poured into a large amount of deionized water. The precipitate was purified by reprecipitation with diethyl ether, then dried under reduced pressure to give PPAA-Val-H as a yellow solid.

### 3.7. Preparation of Chiral Stationary Phases (CSPs)

The poly(phenylacetylene) derivatives (PPAA-Val and PPAA-Val-H) (0.2 g each) were first dissolved in a coating solvent (5 mL), then coated on aminopropyl silanized silica gel (0.8 g) according to a previous method [46]. The coating solvents were THF and *N*,*N*′-dimethylformamide (DMF) and the weight ratio of the polymers to silica gel was 1:4. The polymer-coated silica gels were then packed in a stainless-steel tube (25 cm × 0.20 cm i.d.) by a slurry method. The plate numbers of the packed columns were 1600–3500 for benzene using a hexane/2-propanol (90/10, *v*/*v*) mixture as the eluent at the flow rate of 0.1 mL/min at 25 °C. The dead time (*t*_0_) of the columns was estimated using 1,3,5-tritert-butylbenzene as the non-retained compound [47].

### 3.8. HPLC Measurement

The retention factor, *k*_1_′ = ((*t*_1_ − *t*_0_)/*t*_0_), is the factor indicating the interaction strength between a CSP and the corresponding enantiomer and can be obtained from its elution time *t*_1_ and the dead time *t*_0_. The *k*_1_′ values varied with the coating solvents of the polymers. The separation factor *α*, which is directly correlated to the chiral recognition ability of the CSPs, is an important factor for evaluating the CSPs. If *α* is equal to 1.00, this means no chiral recognition, and the higher the *α* value, the better the chiral recognition ability of the CSPs.

## 4. Conclusions

A phenylacetylene linked with an l-valine ethyl ester group through an amide linkage was synthesized and polymerized to produce a novel one-handed helical *cis*-poly(phenylacetylene) derivative, PPAA-Val. PPAA-Val showed a unique temperature-triggered helix-helix inversion in chloroform without the assistance of extraneous molecules, while it was not observed in a highly polar solvent, such as DMF. This helix-helix inversion is reversible, even after six cycles of a heating-cooling treatment, and the helix sense of the PPAA-Val backbone was still switchable without any loss of the CD intensity. A novel CSP for HPLC prepared by coating PPAA-Val on silica gel particles showed a high chiral recognition ability for racemic mandelonitrile (*α* = 2.18) and racemic *trans*-*N*,*N′*-diphenylcyclohexane-1,2-dicarboxamide (*α* = 2.60). The coating solvent clearly influenced the chiral recognition ability of the CSP; the CSP, coated using DMF as the coating solvent, showed the best chiral recognition. The one-handed helical *cis*-polyene backbone of PPAA-Val was destroyed by heat treatment at 150 °C to afford PPAA-Val-H. Although PPAA-Val-H had the same l-valine ethyl ester pendants as PPAA-Val, PPAA-Val-H did not show any chiral recognition. It was concluded that the one-handed helical *cis*-polyene backbone of the polymer plays a key role in its chiral recognition. In addition, the temperature triggered helix-helix inversion of PPAA-Val may be affected by the molecular weight of the polymer. The research regarding the dependence of the molecular weight on the helix-helix inversion of PPAA-Val will be studied in the near future.

## Figures and Tables

**Table 1:** Specific optical rotations of PAA-Val and PPAA-Val in different solvents.

Compound	[$\alpha_{D}^{20}$]/° ^a^					
	CHCl_3_	MeOH	DMF	DMAc	THF	Acetone
PAA-Val	+57.6	+10.3	+40.6	+40.0	+25.3	+39.3
PPAA-Val	+433	+334	+515	+453	+557	+457

^a^ Measured by polarimetry at room temperature (*c* = 1 mg/mL).

**Table 2:** ν_C=O_ of PAA-Val and PPAA-Val measured in CHCl_3_ and solid state ^a^.

Sample	Condition	Wavenumber (cm^−1^)	
		ν_C=O_ (Amide)	ν_C=O_ (Ester)
**PAA-Val**	CHCl_3_	1662	1735
	solid	1644	1734
**PPAA-Val**	CHCl_3_	1648	1735
	solid	1644	1734

^a^ IR spectra were taken at room temperature in solution (20 mM) or in solid state.

**Table 3:** Resolution of racemates on PPAA-Val and PPAA-Val-H ^a^.

Racemates	PPAA-Val						PPAA-Val-H	
	Coating solvent: DMF		Coating Colvent: THF		Coating Solvent: CHCl_3_		Coating Solvent: DMF	
	*k*_1_′	*α*	*k*_1_′	*α*	*k*_1_′	*α*	*k*_1_′	*α*
**1**	0.19	1.00	0.05	1.00	0.13	1.00	0.11	1.00
**2**	0.44 (−)	2.18	0.13 (−)	2.15	0.24	1.00	1.14	1.00
**3**	0.65	1.00	0.18	1.00	0.33	1.00	0.36	1.00
**4**	1.67	1.00	0.58	1.00	0.84	1.00	0.99	1.00
**5**	13.54 (+)	~1	13.12 (+)	~1	4.33	1.00	5.80	1.00
**6**	0.36 (−)	2.60	0.12 (−)	2.25	0.29 (−)	2.01	0.92	1.00
**7**	4.83	1.00	1.58	1.00	2.18	1.00	1.99	1.00
**8**	2.26	1.00	0.67	1.00	1.12	1.00	1.19	1.00

^a^ Column: 25 cm × 0.2 cm i.d.; Eluent:hexane-2-propanol (90/10, *v*/*v*); Flow rate: 0.1 mL/min; The signs in parentheses represent the optical rotation of the first-eluted enantiomer.
